# Supplementary material for: CYP2C19 Genetic Variants and Major Depressive Disorder: A Systematic Review
Source: Pharmaceuticals (Basel). 2024 Oct 31;17(11):1461. doi: 10.3390/ph17111461 (PMC11597809; doi:10.3390/ph17111461)
Supplement: Supplementary file 1 [file pharmaceuticals-17-01461-s001.zip › pharmaceuticals-3210078-supplementary.pdf]

pharmaceuticals

MDPI

Table S1. Excluded studies and the reason for their exclusion.

Author	Year	Title	Country	Reason for exclusion
Huezo-Diaz et al. [29]	2011	*CYP2C19* genotype predicts steady state escitalopram concentration in GENDEP	Belgium, Croatia, Denmark, England, Germany, Italy, Poland, Slovenia, United Kingdom	Incomplete data
Honeycutt et al.[65]	2022	A Double-Blind Randomized Trial to Investigate Mechanisms of Antidepressant-Related Dysfunctional Arousal in Depressed or Anxious Youth at Familial Risk for Bipolar Disorder	United States	Inadequate study type
Kanders et al. [57]	2020	A pharmacogenetic risk score for the evaluation of major depression severity under treatment with antidepressants	Switzerland	Incomplete data
Sim et al. [47]	2010	Association between *CYP2C19* polymorphism and depressive symptoms	Sweden	Inadequate population
Schenk et al. [66]	2008	Association of graded allele-specific changes in *CYP2D6* function with imipramine dose requirement in a large group of depressed patients	Netherlands	Incomplete data
Ji et al. [67]	2014	Citalopram and escitalopram plasma drug and metabolite concentrations: genome-wide associations	United States	Incomplete data
Veldic et al. [50]	2019	Cytochrome P450 2C19 Poor Metabolizer Phenotype in Treatment Resistant Depression: Treatment and Diagnostic Implications	United States	Incomplete data
Serretti et al. [61]	2009	Cytochrome P450 *CYP1A2*, *CYP2C9*, *CYP2C19* and *CYP2D6* genes are not associated with response and remission in a sample of depressive patients	Belgium, Israel, Austria	Incomplete data
Zastrozhin et al. [59]	2021	Effects of *CYP2C19* genetic polymorphism on the steady-state concentration of citalopram in patients with major depressive disorder	Russia	Inadequate population
Morinobu et al. [28]	1997	Effects of genetic defects in the *CYP2C19* gene on the N-demethylation of imipramine, and clinical outcome of imipramine therapy	Japan	Incomplete data
Matthaei et al. [68]	2021	Effects of Genetic Polymorphism in *CYP2D6*, *CYP2C19*, and the Organic Cation Transporter *OCT1* on Amitriptyline Pharmacokinetics in Healthy Volunteers and Depressive Disorder Patients	Germany	Incomplete data
Arias et al. [69]	2005	Evidence for a combined genetic effect of the 5-HT1A receptor and serotonin transporter genes in the clinical outcome of major depressive patients treated with citalopram	Spain	Incomplete data
Tsai et al. [27]	2010	Genetic polymorphisms of cytochrome P450 enzymes influence metabolism of the antidepressant escitalopram and treatment response	China	Incomplete data
Barthi et al. [70]	2024	Impact of Cytochrome P450 Genetic Variation on Patient-Reported Symptom Improvement and Side Effects Among Children and Adolescents Treated with Fluoxetine	Canada	Incomplete data
Zastrozhin et al. [71]	2021	Impact of the Omics-Based Biomarkers on the Fluvoxamine's Steady-State Concentration, Efficacy and Safety in Patients with Affective Disorders Comorbid with Alcohol Use Disorder	Russia	Inadequate population
Ruaño et al. [72]	2008	Increased carrier prevalence of deficient *CYP2C9*, *CYP2C19* and *CYP2D6* alleles in depressed patients referred to a tertiary psychiatric hospital	United States	Incomplete data
Zastrozhin et al. [54]	2022	Influence of *CYP2C19**17 Genetic Polymorphism on the Steady-State Concentration of Escitalopram in Patients with Recurrent Depressive Disorder	Russia	Inadequate population
Calabrò et al. [26]	2022	Metabolizing status of *CYP2C19* in response and side effects to medications for depression: Results from a naturalistic study	Unidentified	Incomplete data
Zhang et al. [21]	2014	Neither Cytochrome P450 Family Genes nor Neuroendocrine Factors could Independently Predict the SSRIs Treatment in the Chinese Han Population	China	Incomplete data
Zhang et al. [22]	2020	No association between *CYP2C19* genetic polymorphism with treatment remission to antidepressant venlafaxine in Han Chinese population	China	Incomplete data
Torrellas et al. [23]	2017	Optimization of Antidepressant Use with Pharmacogenetic Strategies	Spain	Incomplete data
Docherty et al. [80]	2020	Pathway-based polygene risk for severe depression implicates drug metabolism in CONVERGE	China	Incomplete data
Ng et al. [62]	2013	Pharmacogenetic polymorphisms and response to escitalopram and venlafaxine over 8 weeks in major depression	Australia and China	Incomplete data
Collins et al. [51]	2020	Pharmacogenetic testing in psychiatric inpatients with polypharmacy is associated with decreased medication side effects but not via medication changes	United States	Incomplete data
Poweleit et al. [73]	2019	Pharmacogenetics of sertraline tolerability and response in pediatric anxiety and depressive disorders	United States	Incomplete data
Athreya et al. [56]	2019	Pharmacogenomics-Driven Prediction of Antidepressant Treatment Outcomes: A Machine-Learning Approach with Multi-trial Replication	United States	Incomplete data
Peters et al. [63]	2008	Pharmacokinetic Genes Do Not Influence Response or Tolerance to Citalopram in the STAR*D Sample	Unidentified	Incomplete data
Ruaño et al. [74]	2011	Physiogenomic analysis of CYP450 drug metabolism correlates dyslipidemia with pharmacogenetic functional status in psychiatric patients	United States	Incomplete data
Roll et al. [75]	2022	Rates of Divergent Pharmacogenes in a Psychiatric Cohort of Inpatients with Depression—Arguments for Preemptive Testing	Germany	Incomplete data
Magalhães et al. [76]	2020	Real-World Clinical Characterization of Subjects With Depression Treated With Antidepressant Drugs Focused on (Non-)Genetic Factors, Pharmacokinetics, and Clinical Outcomes: GnG-PK/PD-AD Study	Portugal	Incomplete data
Menke et al. [77]	2020	Roadmap for Routine Pharmacogenetic Testing in a Psychiatric University Hospital	Germany	Incomplete data
Taranu et al. [64]	2017	Should a routine genotyping of *CYP2D6* and *CYP2C19* genetic polymorphisms be recommended to predict venlafaxine efficacy in	France	Incomplete data

		depressed patients treated in psychiatric settings?		
Höfer et al. [78]	2013	The impact of Cytochrome P450 *CYP1A2*, *CYP2C9*, *CYP2C19* and *CYP2D6* genes on suicide attempt and suicide risk—a European multicentre study on treatment-resistant major depressive disorder	Austria, Israel, Belgium, France, Italy	Incomplete data
Ramaraj et al. [24]	2023	The Utility of *CYP2D6* and *CYP2C19* Variants to Guide Pharmacological Treatment in Complex Unipolar Major Depression: A Pilot Longitudinal Study	United Arab Emirates	Incomplete data
Ariefdjohan et al. [79]	2021	The Utility of Pharmacogenetic-Guided Psychotropic Medication Selection for Pediatric Patients: A Retrospective Study	Unidentified	Incomplete data
Hall-Flavin et al. [52]	2013	Utility of integrated pharmacogenomic testing to support the treatment of major depressive disorder in a psychiatric outpatient setting	United States	Incomplete data

Table S2. The most common *CYP2C19* gene polymorphisms' genotype and allele frequency in the studied populations with Major Depressive Disorder (MDD).

MDD		Genotype Frequency										Allele Frequency			
		*1/*1	*1/*2	*2/*2	*1/*3	*3/*3	*2/*3	*1/*17	*17/*17	*2/*17	*3/*17	*1	*2	*3	*17
Asian Continent	Zhang et al.# 2023 [41] (China)											0	0	0	0
American Continent	Islam et al. 2024 [42] (Canada)	71 (40.11%)	39 (22.03%)	3 (1.70%)	1 (0.57%)		2 (1.13%)	43 (24.29%)	7 (3.96%)	10 (5.65%)	1 (0.57%)	225 (63.56%)	57 (16.10%)	4 (1.13%)	68 (19.21%)
	Kharasch et al. 2024 [45] (United States)	26 (38.81%)	12 (17.91%)	3 (4.48%)				21 (31.34%)	2 (2.99%)	3 (4.48%)		85 (63.43%)	21 (15.67%)	0 (0%)	28 (20.90%)
	Montané et al. 2018 [38] (Trinidad and Tobago) Afro-T	8 (40.00%)	8 (40.00%)	1 (5.00%)				2 (10.0%)		1 (5.00%)		26 (65.00%)	11 (27.50%)	0 (0%)	3 (7.50%)
	Montané et al. 2018 [38] (Trinidad and Tobago) Indo-T[a]	7 (17.95%)	11 (28.21%)	7 (17.95%)				6 (15.38%)		7 (17.95%)		31 (41.03%)	32 (41.03%)	0 (0%)	13 (16.61%)
European Continent	Hahn et al. 2021 [25] (Germany)	35 (32.00%)	16 (15.00%)	2 (2.00%)				32 (30.00%)	10 (9.00%)	13 (13.00%)		118 (54.62%)	33 (15.28%)	0 (0%)	65 (30.09%)
	Swiechowski et al. 2021 [39] (Poland)	77 (74.80%)	23 (22.30%)	3 (2.90%)								177 (85.93%)	29 (14.08%)	0 (0%)	0 (0%)
	Jokovićz et al. 2022 [40] (Serbia)	41 (40.20%)	16 (15.69%)	2 (19.61%)				31 (30.00%)	6 (5.88%)	6 (5.88%)		129 (63.24%)	26 (12.75%)	0 (0%)	49 (24.02%)
Transcontinental (Asian/European) Continent	Uckun et al 2015 [36] (Turkey)	24 (48.00%)	7 (14.00%)	1 (2.00%)				16 (32.00%)		2 (4.00%)		71 (71.00%)	11 (11.00%)	0 (0%)	18 (18.00%)
	Yuce-Artun et al 2016 [37] (Turkey)	22 (44.00%)	12 (24.00%)	1 (2.00%)				11 (22.00%)		4 (8.00%)		67 (67.00%)	18 (18.00%)	0 (0%)	15 (15.00%)

Note: # Zhang et al. 2023 (China) is missing as they only informed their gene-level genetic variants description.; [a] Plus the 1.28% from *4 allele frequence.

Table S3. The most common *CYP2C19* gene polymorphisms' phenotypic frequency in the studied populations with Major Depressive Disorder (MDD).

		Phenotype Frequency						
		NM	IM	PM	RM	UM	IM&PM (SM)	RM&UM (FM)
Asian Continent	Zhang et al. 2023 [41] (China)	187 (42.60%)	195 (44.42%)	57 (12.98%)			252 (57.40%)	
American Continent	Islam et al. 2024 [42] (Canada)	71 (40,11%)	51 (28.81%)	5 (2.83%)	43 (24.29%)	7 (3.96%)	56 (31.64%)	50 (28.25%)
	Kharasch et al. 2024 [45] (United States)	26 (38.81%)	15 (22.39%)	3 (4.48%)	21 (31.34%)	2 (2.99%)	18 (26.87%)	23 (34.33%)
	Montané et al. 2018 [38] (Trinidad and Tobago) Afro-T	8 (40.00%)	9 (45.00%)	1 (5.00%)	2 (10.00%)		10 (50.00%)	2 (10.00%)
	Montané et al. 2018 [38] (Trinidad and Tobago) Indo-T	7 (17.95%)	18 (46.15%)	8 (20.51%)	6 (15.38%)		26 (66.66%)	6 (15.38%)
European Continent	Hahn et al. 2021 [25] (Germany)	35 (32.00%)	29 (27.00%)	2 (2.00%)	32 (30.00%)	10 (9.00%)	31 (29.00%)	42 (39.00%)
	Swiechowski et al. 2021 [39] (Poland)	77 (74.80%)	23 (22.30%)	3 (2.90%)			26 (25.20%)	
	Jokovićz et al. 2022 [40] (Serbia)	41 (40.20%)	22 (21.57%)	2 (1.95%)	6 (5.88%)	31 (30.39%)	24 (23.53%)	37 (36.27%)
Transcontinental (Asian/European)	Uckun et al 2015 [36] (Turkey)	24 (48.00%)	9 (18.00%)	1 (2.00%)	16 (32.00%)		10 (20.00%)	16 (32.00%)

Continent	Yuce-Artun et al 2016 [37] (Turkey)	22 (44.00%)	16 (32.00%)	1 (2.00%)	11 (22.00%)		17 (34.00%)	11 (22.00%)

Caption: IM = Intermediate Metabolizer; NM = Normal Metabolizer; PM = Poor Metabolizer; SM = Slow metabolizer (PM&IM); RM = Rapid Metabolizer; UM = Ultrarapid Metabolizer; FM = Fast Metabolizer (RM&UM).

Table S4. Quality Evaluation of articles according to the adapted GRIPS guideline.

**Section/Topic**		**Uckun et al, 2015** [36]	**Yuce-Artun et al, 2016** [37]	**Montané et al, 2018** [38]	**Hahn et al, 2021** [25]	**Świechowski et al, 2021** [39]	**Jоković et al, 2022** [40]	**Zhang et al, 2023** [41]	**Islam et al, 2024** [42]	**Kharasch et al, 2024** [45]
Methods										
Study design and setting	4) Present key elements of study design early in the paper and describe the setting, locations, and relevant dates, including periods of recruitment, exposure, follow-up, and data collection.	x	x	x	x	x	x	x	x	x
Participants	5) Describe eligibility criteria for participants, and sources and methods of selection of participants.	x	x	x	NA	x	x	x	x	x
Variables: Definition	6 (a) Clearly define all participant characteristics	x	x	x	x	x	x	x	x	x
	6 (b) Clearly define risk factors	NA	NA	NA	NA	NA	NA	x	NA	NA
	6 (c) Clearly define outcomes.	x	x	x	x	x	x	x	x	x
	6 (d) Clearly define genetic variants using a widely-used nomenclature system.	x	x	x	x	x	x	x	x	x
Variables: Assessment	7) (a) Describe sources of data and details of methods of assessment (measurement) for each variable.	x	x	x	x	x	x	x	x	x
	7 (b) Give a detailed description of genotyping and other laboratory methods.	x	x	x	x	x	x	x	x	x
Variables: Coding	8) (a) Describe how genetic variants were handled in the analyses	x	x	x	x	x	x	x	x	x
	8 (b) Explain how other quantitative variables were handled in the analyses. If applicable, describe which groupings were chosen, and why.	x	x	x	x	x	x	x	x	x
Analysis: Risk model construction	9) Specify the procedure and data used for the derivation of the risk model. Specify which candidate variables were initially examined or considered for inclusion in models. Include details of any variable selection procedures and other model building issues. Specify the horizon of risk prediction (e.g., 5-year risk).	NA	NA	NA	NA	NA	NA	x	NA	NA
Analysis: Validation	10) Specify the procedure and data used for the validation of the risk model.	NA	NA	NA	NA	NA	NA	x	NA	NA
Analysis: Missing data	11) Specify how missing data were handled.	x	NA	x	NA	NA	x	NA	x	x
Analysis: Statistical methods	12) Specify all measures used for the evaluation of the risk model including, but not limited to, measures of model fit and predictive ability.	NA	NA	NA	NA	NA	NA	x	NA	NA
Analysis: Other	13) Describe all subgroups, interactions, and exploratory analyses that were examined.	x	x	x	x	x	x	x	x	x
Results										
Participants	14) Report the numbers of individuals at each stage of the study. Give reasons for nonparticipation at each stage. Report the number of participants not genotyped,	x	x	x	x	x	x	x	x	x

	and reasons why they were not genotyped.									
Descriptives: Population	15) (a) Report demographic and clinical characteristics of the study population	x	x	x	x	x	x	x	x	x
	15) (b) Report demographic and clinical characteristics of the study population, including risk factors used in the risk modeling.	NA	NA	NA	NA	NA	NA	x	NA	NA
Descriptives: Model estimates	16) Report unadjusted associations between the variables in the risk model(s) and the outcome. Report adjusted estimates and their precision from the full risk model(s) for each variable.	NA	NA	NA	NA	NA	NA	x	NA	NA
Risk distributions	17) Reports the predicted risk distribution and/or its scores.	NA	NA	NA	NA	NA	NA	x	NA	NA
Assessment	18) Report measures of model fit and predictive ability, and any other performance measures, if pertinent.	NA	NA	NA	NA	NA	NA	x	NA	NA
Validation	19) Report any validation of the risk model(s).	NA	NA	NA	NA	NA	NA	x	NA	NA
Other analyses	20) Present results of any subgroup, interaction, or exploratory analyses, whenever pertinent.	x	x	x	x	x	x	x	x	x
Discussion										
Limitations	21) Discuss limitations and assumptions of the study, particularly those concerning study design, selection of participants, and measurements and analyses, and discuss their impact on the results of the study.	x		x		x	x	x	x	x
Interpretation	22) Give an overall interpretation of results considering objectives, limitations, multiplicity of analyses, results from similar studies, and other relevant evidence.	x	x	x	x	x	x	x	x	x
Generalizability	23) Discuss the generalizability and, if pertinent, the health care relevance of the study results.	x	x	x	x	x	x	x	x	x

X = Present; NA = not applicable.

Table S5. Quality Evaluation of articles according to the adapted STROPS guideline.

**Section/Topic**		**Uckun et al, 2015** [36]	**Yuce-Artun et al, 2016** [37]	**Montané et al, 2018** [38]	**Hahn et al, 2021** [25]	**Świechowski et al, 2021** [39]	**Joković et al, 2022** [40]	**Zhang et al, 2023** [41]	**Islam et al, 2024** [42]	**Kharasch et al, 2024** [45]
Methods										
Study design	6) Present key elements of study design early in the paper.	x	x	x	x	x	x	x	x	x
Setting	7) Describe the setting, locations and relevant dates, including periods of recruitment, follow-up, and data collection.	x	x	x	x	x	x	x	x	x
Participants	8) Give the eligibility criteria, and the sources and methods of selection of participants. For a cohort study, describe methods of follow-up. For a case-control study, state whether true controls or population controls were used. Give the rationale for the choice of cases and controls.	x	x	x	NA	x	x	x	x	x
	9) Report the drug and regime participants were exposed to, and the length of exposure.	x	x	x	NA	x	NA	NA	x	x
	10) For a matched case-control study, give matching criteria and the number of controls per case.	x	NA	NA	NA	x	NA	x	NA	NA
	11) Give information on the criteria and methods for selection of subsets of participants from a larger study, when relevant.	x	x	x	x	x	x	x	x	x
	12) If other publications report results for the same patient cohort, or a subset of the patient cohort, provide information on this patient cohort overlap and references to the relevant publications.	NA	NA	NA	NA	NA	NA	NA	x	x
	13) Report disease/clinical indication of patients using a standardised ontology when possible.	x	x	x	x	x	x	x	x	x
Variables	14) Clearly define all outcomes, potential	x	x	x		x	x	x	x	x

	confounders, and effect modifiers. Give diagnostic criteria, if applicable.									
	15) Provide justification for choice of outcomes.	x	x	x	x	x	x	x	x	x
	16) Clearly define genetic exposures (genetic variants) using a widely-used nomenclature system.	x	x	x	x	x	x	x	x	x
	17) Report the rs number of each genotyped SNP.	x	x	x	x	x	x	x	x	x
	18) Clearly state how haplotypes or star alleles were defined.	x	x	x	x	x	x	x	x	x
	19) If referring to the minor, major, wild-type, mutant, reference, risk or effect allele of a variant, state which allele this is and for which given population/cohort.	x	x	x	x	x	x	x	x	x
Data sources/ measurement	20) For each variable of interest, give sources of data and details of methods of assessment (measurement). Describe comparability of assessment methods if there is more than one group.	x	x	x	x	x	x	x	x	x
	21) Describe laboratory methods, including source and storage of DNA, genotyping methods and platforms (including the allele calling algorithm used, and its version), error rates and call rates. State the laboratory/centre where genotyping was done. Describe comparability of laboratory methods if there is more than one group. Specify whether genotypes were assigned using all of the data from the study simultaneously or in smaller batches.	x	x	x	x	x	x	x	x	x
	22) Describe genotype quality control methods and findings.					x				
	23) For quantitative outcome variables, specify if any investigation of potential bias resulting from pharmacotherapy was undertaken. If relevant, describe the nature and magnitude of the potential bias, and explain what approach was used to deal with this.	x	x	x	NA	NA	x	NA	x	
	24) Report how adherence to treatment was assessed, and report the results of the assessment.			x	NA		x	NA	x	
Study size	25) Explain how the study size was arrived at, or provide details of the a priori power to detect effect sizes of varying degrees.						x			
Quantitative variables	26) Explain how quantitative variables (confounders and effect modifiers) were handled in the analyses. If applicable, describe which groupings were chosen, and why.	x	x	x	NA	x	x	x	x	x
Statistical methods	27) (a) Describe methods used to control for confounding	x	x	x		x	x	x	x	
	27) (b) Describe any methods used to examine subgroups and interactions.	x	x	x	x	x	x	x	x	x
	27) (c) Explain how missing data were addressed.	x	NA	x	NA	NA	x	NA	x	x
	27) (d) Cohort study – If applicable, explain how loss to follow-up was addressed.	NA	NA	x	NA	NA	x	NA	x	x
	27) (e) Case-control study – If applicable, explain how matching of cases and controls was addressed.	x	NA	NA	NA	x	NA	x	NA	NA
	27) (f) Describe any sensitivity analyses.				NA		x	NA		
	28) State whether Hardy-Weinberg equilibrium was considered and, if so, how.	x				x		x	x	
	29) Describe any methods used for inferring genotypes or haplotypes.	x	x	x	x	x	x	x	x	x
	30) Describe any methods used to assess or address population stratification.	x	x	x	x	x	x	x	x	x
	31) Describe any methods used to assess and correct for relatedness among subjects. Report results of assessments for relatedness.	x								
	32) Describe any methods used to address multiple comparisons or to control risk of false positive results due to a) multiple genetic variants b) multiple outcomes c) multiple assumptions regarding mode of inheritance	x	x	x	x	x	x	x	x	x
	33) Describe any methods used to adjust for extent of adherence in the analyses.	NA	NA	x	NA		x	NA	x	
Results										
Participants	34) Report the numbers of individuals at each stage of the study – e.g., numbers potentially eligible, examined for eligibility, confirmed eligible, included in the study, completing follow-up, and analysed.	x	x	x	x	x	x	x	x	x
SNPs	35) Report any SNPs that were excluded from analysis, and provide reasons for these exclusions.	x	NA	NA	NA	NA	NA	NA	NA	NA
Descriptive data	36) Give characteristics of study participants (e.g., demographic, clinical, social, ethnicity) and information on potential confounders.	x	x	x	x	x	x	x	x	x
	37) Cohort study – Summarize follow-up time, e.g. average and/or total amount.	NA	NA	x	NA	NA	x	NA	x	x
	38) Where HWE tests have been undertaken, highlight SNPs that deviate from HWE.	x				X		x	x	
	39) Where population stratification is assessed, report	x	x	x	x	X	x	x	x	x

	the results.									
Outcome data	40) (a) For a cohort study, report all outcomes (phenotypes) investigated for each genotype category over time.	NA	NA	x	NA	NA	x	NA	x	x
	40) (b) For a case-control study, report numbers in each genotype category for all outcomes investigated.	x	NA	NA	NA	x	NA	x	NA	NA
	40) (c) For a cross sectional study, report all outcomes (phenotypes) investigated for each genotype category.	NA	x	NA	x	NA	NA	NA	NA	NA
	41) If a study includes more than one ethnic group, provide the summary data specified in (40) per ethnic group.	NA	NA	x	NA	NA	NA	NA	x	x
Main results	42) Give unadjusted estimates and, if applicable, confounder-adjusted estimates and their precision (e.g., 95% confidence intervals). Make clear which confounders were adjusted for and why they were included.	x	x				x	x	x	
	43) Report category boundaries when continuous variables were categorised.	NA	NA	NA	NA	NA	NA	NA	NA	NA
Other analyses	44) Report other analyses done – e.g., analyses of subgroups and interactions, and sensitivity analyses.	x	x	x		x	x	x	x	x
	45) If numerous genetic exposures (genetic variants) were examined, summarize results from all analyses undertaken.	x	x	x	x	x	x	x	x	x
	46) If detailed results are available elsewhere, i.e. in supplementary materials, state how they can be accessed.	NA	NA	x	NA	NA	x	NA	x	x
Discussion										
Key results	47) Summarize key results with reference to study objectives.	x	x	x	x	x	x	x	x	x
Limitations	48) Discuss limitations of the study, taking into account sources of potential bias or imprecision. Discuss both direction and magnitude of any potential bias.	x		x		x	x	x	x	x
Interpretation	49) Give a cautious overall interpretation of results considering objectives, limitations, multiplicity of analyses, results from similar studies, and other relevant evidence.	x	x	x	x	x	x	x	x	x
Generalisability	50) Discuss the generalisability (external validity) of the study results.	x	x	x	x	x	x	x	x	x
Other information										
Study registration	51) State whether the study has been registered. If the study has been registered, provide details of the registry.	NA	NA	NA	NA	NA	NA	NA	x	x
Ethical approval	52) Report whether ethical approval was obtained for the collection of genetic data.	x	x	x	x	x	x	x	x	x
Funding	53) Give the source of funding and the role of the funders for the present study and, if applicable, for the original study on which the present article is based.	x	x	x		x	x	x	x	x
Databases	54) State whether databases for the analysed data are or will become publicly available and if so, how they can be accessed.	NA	NA	x		NA	NA	x	x	x

X = Present; NA = not applicable.
